# Supplementary material for: The effects of genital myiasis on the diversity of the vaginal microbiota in female Bactrian camels
Source: BMC Vet Res. 2022 Mar 5;18:87. doi: 10.1186/s12917-022-03189-5 (PMC8897907; doi:10.1186/s12917-022-03189-5)
Supplement: Supplementary file 5 — Additional file 5. [file 12917_2022_3189_MOESM5_ESM.zip › MPL201709200_16s_yy/Treat1/B13_bdiv/weighted_unifrac_2d_continuous/weighted_unifrac_pc_2D_PCoA_plots.html]

20\_bdiv/weighted\_unifrac\_2d\_continuous/weighted\_unifrac\_pc\_2D\_PCoA\_plots.html


| Treat1 | | |
| --- | --- | --- |
| Download Figure | Download Figure | Download Figure |
